# Left Atrial Appendage Occlusion with the Watchman FLX Device: A Single-Centre Retrospective Analysis of Procedural and One-Year Clinical Outcomes

**DOI:** 10.3390/medsci14060665

**Published:** 2026-10-12

**Authors:** Wasim Allakkis, Mostafa Salem, Mohamed Salem, Mohamed Elabd, Ali Balakishiyev, Moussab Aboud, Ammar Fayoud, Mohammed Saad, Mohmmad Hamadneh

**Affiliations:** 1 Department of Cardiology, Helios Frankenwaldklinik-Hospital, 96317 Kronach, Germany; 2 Department of Cardiology, University Hospital Schleswig-Holstein, 24105 Kiel, Germany; 3 Department of Congenital Heart Surgery and Congenital Heart Defects, Pediatric Heart Centre, University Hospital of Giessen and Marburg, 35392 Giessen, Germany; 4 Department of Cardiology, Sana Hospital, 96450 Coburg, Germany

**Keywords:** left atrial appendage occlusion, Watchman FLX, atrial fibrillation, stroke, mortality

## Abstract

**Background:** Left atrial appendage occlusion (LAAO) is an alternative approach to stroke prevention in selected patients with non-valvular atrial fibrillation. This single-centre retrospective study describes device implantation, peri-procedural complications and clinical outcomes during the first year after Watchman FLX implantation. **Methods:** We analysed 100 consecutive patients who underwent LAAO with the Watchman FLX device at Helios Frankenwaldklinik-Hospital, Kronach, Germany, between 2021 and 2024. The primary endpoint was device implantation success, defined as correct device deployment and implantation. The secondary safety endpoint was a composite of major peri-procedural complications within 72 h. Additional clinical outcomes were strokes and mortality at 30 days and one year. **Results:** The mean age was 70.7 ± 6.5 years, and 47% of patients were female. The mean CHA_2_DS_2_-VASc score was 4.9 ± 1.4, and the mean HAS-BLED score was 4.1 ± 1.0. Device implantation was successful in 100% (95% CI 96.4–100%). The reported peri-procedural complication rate was 7% (95% CI 2.9–13.9%), including major bleeding in 6% (95% CI 2.2–12.6%) and pericardial tamponade in 1% (95% CI 0.0–5.4%). Thirty-day mortality was 2% (95% CI 0.2–7.0%), with no reported strokes (0%; 95% CI 0.0–3.6%). One-year mortality was 11% (95% CI 5.6–18.8%), with one reported stroke (1%; 95% CI 0.0–5.4%). **Conclusions:** Watchman FLX implantation was achieved in all patients in this cohort. Bleeding was the most frequently reported peri-procedural complication. The low observed stroke proportion should be interpreted alongside the 11% one-year mortality and incomplete imaging surveillance. This uncontrolled descriptive study cannot establish comparative efficacy or comprehensive device safety.

## 1. Introduction

Atrial fibrillation (AF) is a common cardiac arrhythmia whose prevalence increases with age. Epidemiological studies describe its substantial global burden [1,2,3]. AF is associated with an increased risk of ischaemic stroke, making stroke prevention a central component of care [4].

Oral anticoagulation (OAC) with vitamin K antagonists (VKAs) or direct oral anticoagulants (DOACs) is an established strategy for stroke prevention in AF [5,6]. In selected patients who cannot receive long-term OAC, percutaneous left atrial appendage occlusion (LAAO) may be considered [7]. The rationale is that the left atrial appendage (LAA) is an important source of thrombus in non-valvular AF [8,9,10]. Several percutaneous devices have been developed to exclude the LAA from the circulation [11,12,13].

The Watchman device (Boston Scientific Corporation, Marlborough, MA, USA) consists of a self-expanding nitinol frame with a fabric covering and is delivered through a transseptal sheath [12]. Post-implantation antithrombotic treatment varies across studies and clinical settings [14]. The pivotal Watchman trials used an initial course of warfarin and aspirin, followed by aspirin and clopidogrel and then aspirin alone [15,16]. This trial regimen differs from the three-month dual antiplatelet protocol used at our centre.

Randomised trials and observational registries, including PROTECT AF, PREVAIL, and EWOLUTION, have evaluated the Watchman device [15,16,17]. These studies provide context for interpreting local outcomes, but differences in device generation, patient selection and outcome assessment limit direct comparisons.

This single-centre retrospective study describes device implantation success, peri-procedural complications, and stroke and mortality outcomes through one year in patients treated with the Watchman FLX device.

## 2. Methods

### 2.1. Study Design and Patient Population

This single-centre, retrospective observational study included 100 consecutive patients who underwent percutaneous LAAO at Helios Frankenwaldklinik Hospital in Kronach, Germany, between 2021 and 2024. All 100 patients received the Watchman FLX device; no patients received Watchman 2.5. We based LAAO indications on guideline and consensus recommendations [7,18]. The study excluded patients younger than 18 years and patients for whom neither the patient nor a legal representative could provide written consent, or patients who would not be able to stop taking OAC for any medical condition. Study planning began before the first implantation so that all patients or their legal guardians had signed the implantation consent as well as a general research consent.

The study period ended in 2024 to allow assessment of clinical outcomes over the year following implantation. From late 2024, our centre adopted an LAAO device from another manufacturer. These procedures were outside the scope of this Watchman-only cohort.

Demographic characteristics, procedural data and clinical outcomes were analysed retrospectively. A clinical event committee of two independent physicians adjudicated adverse events, with a third physician consulted in cases of disagreement.

On 26 March 2026, the Ethics Committee of the Bavarian State Medical Association classified this project as a retrospective quality-control study and determined that it was not subject to mandatory consultation under Section 15 of the Professional Code for Physicians in Bavaria (reference 26008-BO). Written informed consent covering both device implantation and the scientific use of clinical data was obtained from all participants or their legal representatives. The study was conducted in accordance with the Declaration of Helsinki.

### 2.2. Data Collection

Demographic and clinical characteristics, procedural details, in-hospital treatment and adverse events were extracted from electronic medical records.

Clinical events up to one year after implantation were ascertained through institutional medical records, discharge reports from other hospitals and telephone contact with patients, relatives or treating physicians. Telephone follow-up covered the entire cohort; some patients also continued to receive outpatient or inpatient care at our institution. Clinical follow-up for vital status and stroke was complete for all 100 patients through one year after implantation or until death; no patients were lost to clinical follow-up. Available information on the date and cause of death was reviewed. Post-procedural imaging was not performed systematically in all patients; an imaging-based analysis of device-related thrombus, peri-device leak and late device position was therefore not performed.

### 2.3. LAAO Procedure

All procedures were performed under conscious sedation with fluoroscopic and transoesophageal echocardiography (TOE) guidance. Transseptal puncture used standard techniques. Watchman FLX size was selected using TOE measurements, with a device diameter 10–20% larger than the maximum LAA landing-zone diameter. Local anaesthesia consisted of 1% mepivacaine at the puncture site. Propofol was administered continuously at the lowest effective dose, and the standard protocol required four hours of post-procedural monitoring. Patients with active infection, pregnancy, an estimated life expectancy below one year, or an indication for OAC other than AF were not considered eligible for implantation.

The institutional post-implantation protocol was aspirin plus clopidogrel for three months, followed by lifelong aspirin monotherapy. Clopidogrel monotherapy was used in patients with aspirin intolerance. This strategy was selected to avoid long-term OAC exposure and reduce the associated bleeding risk in patients considered at high bleeding risk; patients with an indication for OAC other than AF were not offered LAAO. Antiplatelet-based treatment in this setting is discussed in the EHRA/EAPCI consensus recommendations [18]. Medication intake was queried during follow-up. The analysis did not quantify the number or timing of treatment deviations, interruptions or subsequent OAC prescriptions. The intended protocol should therefore be distinguished from uninterrupted treatment exposure in every patient, particularly in those who died during follow-up.

### 2.4. Study Endpoints

The primary endpoint was device implantation success, defined as correct deployment and implantation of the Watchman FLX device in the intended LAA position. This endpoint did not require freedom from peri-procedural complications. LAAO consensus definitions distinguish device deployment, technical success including adequate LAA occlusion, and procedural success including freedom from major complications [19]. The implantation endpoint reported here should not be interpreted as a complete consensus-defined technical or procedural success endpoint; these broader endpoints were not calculated.

The secondary safety endpoint was a composite of major peri-procedural complications occurring within 72 h of the index procedure: death, stroke, device embolisation, cardiac tamponade or pericardial effusion requiring intervention, major bleeding (BARC type > 3a), bailout surgery, cardiopulmonary resuscitation, and other major cardiac or vascular injuries. Additional clinical outcomes were stroke, all-cause mortality and cardiovascular mortality at 30 days and one year.

Bleeding terminology and cardiovascular event classification were informed by published consensus definitions [20,21,22]. The study-specific endpoint definition and 72-h observation window are stated explicitly because definitions and assessment periods differ across LAAO studies. The composite safety endpoint is defined at patient level; individual component categories need not be mutually exclusive.

### 2.5. Statistical Analysis

This study is descriptive. Continuous variables are summarised as mean ± standard deviation (SD), and categorical variables as counts and percentages. Exact (Clopper–Pearson) 95% confidence intervals (CIs) accompany principal outcome proportions. Follow-up outcomes are reported as observed events divided by the original cohort of 100 patients, rather than person-time incidence rates or competing-risk estimates. No patient-level comparisons of survivors and decedents or patients with and without complications, and no multivariable models, were performed. The small number of events, including 11 deaths, limits reliable adjustment for the several proposed predictors. Exploratory two-sided exact binomial tests compare the observed counts with selected published rates treated as fixed reference probabilities. These are one-sample benchmark tests, not formal between-cohort tests; they do not account for uncertainty in the reference estimate, differences in follow-up, baseline risk or multiple comparisons. They cannot establish superiority, non-inferiority or equivalence. Descriptive analyses used GraphPad Prism 10 (GraphPad Software, Boston, MA, USA); confidence intervals and the revised benchmark tests were independently verified using Python 3.12.14.

## 3. Results

### 3.1. Patient Characteristics

All 100 patients received the Watchman FLX device. Baseline characteristics are summarised in Table 1.

**Figure 1:**
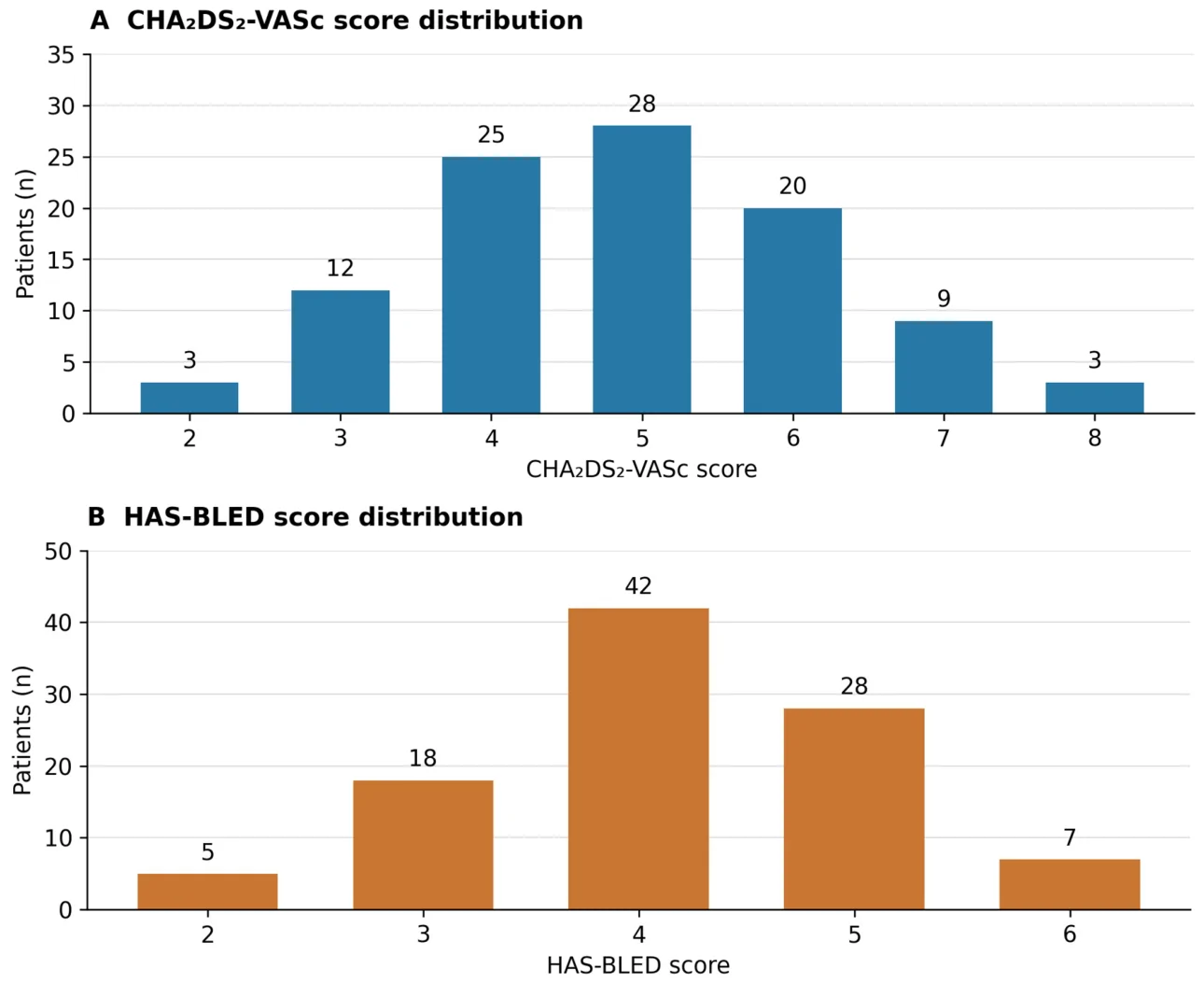
(**A**) Distribution of CHA_2_DS_2_-VASc scores in the study cohort (*n* = 100). Bars show the number of patients in each score category. (**B**) Distribution of HAS-BLED scores in the study cohort (*n* = 100). Bars show the number of patients in each score category.

The cohort’s mean age was 70.7 ± 6.5 years and 47% were female. Most participants had arterial hypertension (88%), coronary artery disease (71%) and chronic kidney disease (40%) at various stages; 5% received regular haemodialysis. The cohort exhibited a high mean stroke risk (mean CHA_2_DS_2_-VASc score 4.9) and a high mean bleeding risk (mean HAS-BLED score 4.1). The score distributions are shown in Figure 1A,B.

### 3.2. Device Implantation Outcomes

Device implantation was successful in all 100 patients (100%; 95% CI 96.4–100%). No device embolisation or implantation failure occurred (0% each; 95% CI 0.0–3.6%).

### 3.3. Peri-Procedural Complications

**Figure 2:**
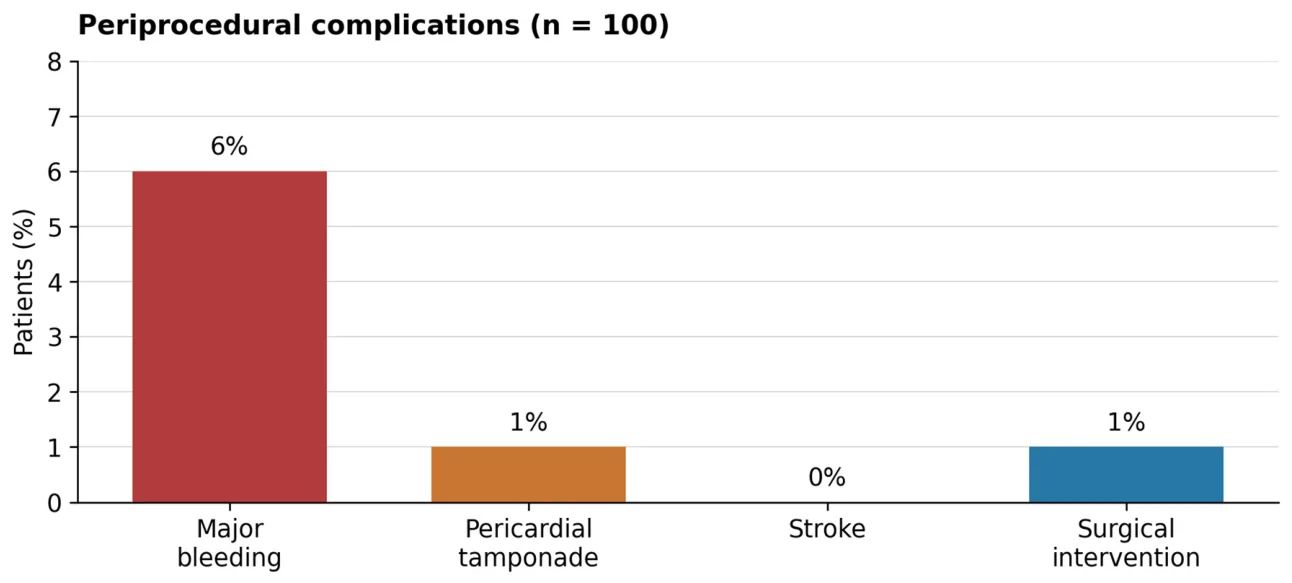
Peri-procedural complication categories within 72 h of implantation in the 100-patient cohort. Bars show the percentage of patients in each category. The surgical intervention occurred in one of the six bleeding patients; tamponade affected a seventh patient. Thus, seven unique patients had at least one complication, and the component percentages should not be added.

Seven patients experienced at least one major peri-procedural complication (7%; 95% CI 2.9–13.9%): six had major bleeding (6%; 95% CI 2.2–12.6%), and one additional patient had pericardial tamponade (1%; 95% CI 0.0–5.4%). The patient requiring surgical intervention (1%; 95% CI 0.0–5.4%) was included among the six patients with major bleeding. No peri-procedural stroke was reported (0%; 95% CI 0.0–3.6%). The individual complication categories are shown in Figure 2.

One of the six patients with major bleeding experienced a post-procedural haematoma in the right groin that required surgical evacuation.

One patient developed intra-procedural cardiac tamponade and underwent successful pericardiocentesis. Surgical intervention was not required. The patient remained haemodynamically stable during subsequent monitoring and was discharged without further complications.

### 3.4. Clinical Outcomes

**Figure 3:**
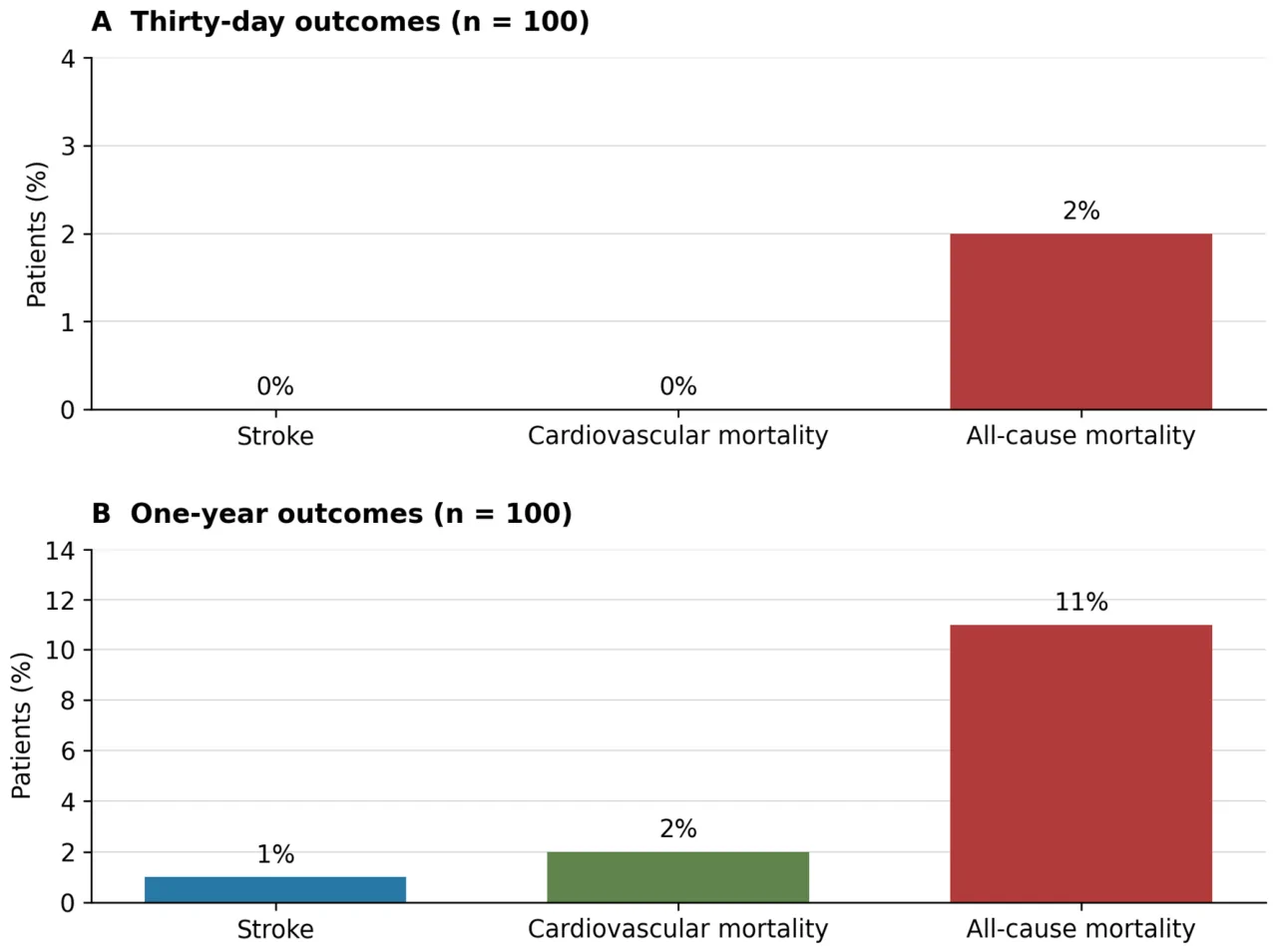
Clinical outcomes after Watchman implantation. Panel (**A**) shows 30-day outcomes; panel (**B**) shows one-year outcomes. Bars show the percentage of the original 100-patient cohort with stroke, cardiovascular death or death from any cause. Cardiovascular deaths are included in all-cause mortality.

Clinical follow-up for vital status and stroke was complete for all 100 patients through one year or until death. Stroke and mortality at 30 days and one year are shown in Figure 3A,B.

Of the 100 patients included in this study, 11 died within one year following the procedure, resulting in an all-cause mortality rate of 11% (95% CI 5.6–18.8%). Two deaths occurred within the first 30 days, corresponding to a 30-day mortality rate of 2% (95% CI 0.2–7.0%). Cardiovascular mortality was 0% at 30 days (95% CI 0.0–3.6%) and 2% at one year (95% CI 0.2–7.0%).

Both 30-day deaths were attributed to non-cardiovascular causes. One patient died from advanced urinary bladder cancer while the other died from end-stage chronic renal failure.

Of the remaining nine deaths occurring between 30 days and one year, two patients died at home at the ages of 88 and 89 years, respectively; the exact causes of death are unknown. Two additional patients died at home due to end-stage renal failure. Other causes of death included decompensated heart failure in two patients, sepsis in one patient, acute renal failure in one patient and pneumonia in one patient.

No strokes were reported within 30 days (0%; 95% CI 0.0–3.6%). One non-disabling stroke was reported within one year (1% of the original cohort; 95% CI 0.0–5.4%).

## 4. Discussion

### 4.1. Principal Findings and Comparison with Previous Studies

In this descriptive cohort, Watchman FLX implantation was achieved in all 100 patients. Major bleeding was the most frequently reported peri-procedural complication. During the first year, one stroke and 11 deaths were reported. These findings describe local clinical experience; they do not demonstrate treatment efficacy or establish that implantation was free of complications.

Table 2 provides a focused comparison with contemporary studies using the same Watchman FLX generation. Published definitions, follow-up completeness and methods of estimating event rates still differ. The exploratory benchmark tests should therefore be read alongside the cohort CIs and should not be used to infer equivalence or a centre-specific advantage.

PROTECT AF, PREVAIL, EWOLUTION and the earlier NCDR registry provide historical context but predominantly concern the earlier Watchman generation [15,16,17,25,26,27]. Their implantation and safety outcomes should not be treated as directly interchangeable with those of this FLX cohort. In particular, comparisons across dissimilar peri-procedural windows and composite endpoints may be misleading.

The 95% CI around our 100% implantation estimate extends down to 96.4%. Successful implantation alone does not demonstrate absence of complications or complete long-term LAA exclusion. Differences in patient selection, operator experience and antithrombotic treatment could influence outcomes across studies, but their contribution was not tested in this cohort.

### 4.2. Peri-Procedural Bleeding and Tamponade

Six patients had reported major bleeding, and one patient required surgical evacuation of an access-site groin haematoma. This makes bleeding an important safety concern despite successful device implantation. The high mean HAS-BLED score and frequent renal disease identify relevant clinical context, but they do not establish why these events occurred or which characteristics predicted them.

Bleeding after LAAO can occur before any longer-term benefit from avoiding anticoagulation is realised. A pooled analysis of the randomised Watchman trials found that bleeding outcomes varied over time and according to exposure to adjunctive antithrombotic therapy [28]. Our retrospective data cannot determine whether a different regimen or access-management strategy would have prevented bleeding. Anatomical site, timing, BARC grade, management and recovery should be documented consistently in future assessments.

The tamponade event resolved after pericardiocentesis. The available account does not establish that propofol exposure or patient movement caused the event. Prior observational work has examined conscious sedation and general anaesthesia for Watchman implantation [29], but this single event cannot guide a comparison of anaesthetic strategies.

### 4.3. Stroke Outcomes and Antithrombotic Treatment

One non-disabling stroke was reported within one year (1%; 95% CI 0.0–5.4%). The wide CI reflects the uncertainty of a single event in a small cohort. CHA_2_DS_2_-VASc describes baseline risk and cannot replace an untreated or anticoagulated control group. We therefore do not infer a treatment effect or a relative stroke-risk reduction from this observation. Death also limits the time during which a patient can experience stroke; no competing-risk analysis was performed.

Our three-month aspirin–clopidogrel protocol was intended to avoid long-term OAC exposure in patients at high bleeding risk. The EHRA/EAPCI consensus describes aspirin with clopidogrel for one to six months after Watchman implantation in this setting, with treatment decisions informed by adequate occlusion and the absence of device-related thrombus [18]. The 2024 international consensus specifically includes three months of aspirin plus clopidogrel after Watchman FLX implantation, followed by aspirin alone [30]. These recommendations provide context for the local strategy; they do not establish it as superior to alternative regimens.

The subsequent 2025 SCAI/HRS guideline conditionally supports either post-procedural OAC or dual antiplatelet therapy, with DAPT a reasonable choice when OAC is unsuitable because of bleeding risk; it makes no recommendation for single antiplatelet therapy because of an evidence gap [31]. Clopidogrel monotherapy for aspirin intolerance represented an individualised local alternative. Medication intake was queried at follow-up, but this does not establish uninterrupted adherence or permit comparison of regimens. Clinical follow-up was complete, whereas imaging surveillance was not systematic. Since device-related thrombus has been associated with embolic events [32], a low clinical stroke proportion cannot exclude subclinical device complications. Neither LAAO nor antiplatelet treatment eliminates bleeding risk.

### 4.4. Mortality and Competing Clinical Risks

Of the 11 deaths reported within one year, two were cardiovascular, seven were attributed to non-cardiovascular causes, and two had an unknown cause (Table 3). We retained the unknown deaths as a separate category. Without additional information, we cannot confidently classify all deaths as unrelated to the device or procedure. When interpreting clinical benefit, consider observed mortality alongside stroke outcomes.

Both deaths within 30 days were attributed to severe pre-existing disease. Their occurrence raises questions about prognosis and patient selection, but does not establish what could reasonably have been predicted at implantation. No comparison of baseline characteristics between decedents and survivors was performed, so greater comorbidity remains a possible explanation rather than a demonstrated cause of mortality. Long-term trial results provide a different time horizon from this one-year cohort [33]. Assessment of expected survival, frailty, renal disease and oncological status is relevant when considering whether a patient may live long enough to benefit from stroke prevention.

### 4.5. Clinical Implications and Future Directions

This series identifies bleeding management, documentation of antithrombotic treatment, follow-up imaging and assessment of prognosis as priorities for local review. It does not determine an optimal medication regimen or anaesthetic approach. Evidence from randomised comparisons, including PRAGUE-17 [34], must be distinguished from the descriptive findings reported here. Larger prospective studies with consistent event definitions, complete follow-up and documented reasons for LAAO are needed to examine treatment effects and predictors of adverse outcomes.

### 4.6. Study Limitations

The retrospective, single-centre design, absence of a control group and small number of events limit generalisability and prevent causal or comparative efficacy conclusions. The reported CIs are wide, and no patient-level risk-factor analyses or multivariable adjustment were performed.

Clinical follow-up for vital status and stroke was complete through one year or until death, but retrospective event ascertainment relied on clinical records and telephone reports rather than uniform prospective assessments. Death was a competing event for stroke, and event-time data were not analysed using a competing-risk method.

Post-procedural imaging was not systematic. Imaging attendance, timing and findings were not quantified, so reliable frequencies of device-related thrombus, peri-device leak and late device malposition cannot be provided. The absence of a reported clinical event should not be interpreted as the absence of an imaging-detectable complication.

Patient-level indications for LAAO and detailed bleeding classifications were not systematically reported in this analysis. Aspirin intolerance led to use of clopidogrel monotherapy, introducing treatment heterogeneity. Medication intake was queried at follow-up, but the number and timing of regimen changes and interruptions were not quantified, and no comparative analysis by treatment regimen was performed. Self-reported intake does not establish uninterrupted adherence. These limitations prevent attribution of outcomes to a uniform antithrombotic regimen or a demonstrated reduction in bleeding risk.

Comparisons with published cohorts remain descriptive because device generations, baseline risk, outcome definitions, follow-up windows and denominators differ. The fixed-rate binomial tests do not address these differences or the uncertainty in the reference estimates. A non-significant result is not evidence of equivalence. Follow-up beyond one year is needed to assess later events.

## 5. Conclusions

In this single-centre cohort of 100 patients, Watchman FLX implantation was achieved in all patients. Major bleeding was the most frequently reported peri-procedural complication. One stroke and 11 deaths were reported during the first year. These observations support the feasibility of implantation in this setting but cannot establish comparative stroke-prevention efficacy, the safety of a specific antithrombotic regimen, or comprehensive device safety without systematic imaging and more complete outcome characterisation.

## Figures and Tables

**Table 1:** Baseline Characteristics (*n* = 100).

Characteristic	Value
Age (years)	70.7 ± 6.5 (range 57–91)
Female, *n* (%)	47 (47%)
Body mass index (kg/m^2^)	28.6 ± 5.5
Arterial hypertension, *n* (%)	88 (88%)
Diabetes mellitus, *n* (%)	38 (38%)
Coronary artery disease, *n* (%)	71 (71%)
Chronic kidney disease, *n* (%)	40 (40%)
Regular haemodialysis, *n* (%)	5 (5%)
Chronic lung disease, *n* (%)	15 (15%)
Chronic liver disease, *n* (%)	11 (11%)
CHA_2_DS_2_-VASc score	4.9 ± 1.4
HAS-BLED score	4.1 ± 1.0

Continuous variables are presented as mean ± SD (range where indicated); categorical variables as *n* (%).

**Table 2:** Selected outcomes in the present cohort and contemporary Watchman FLX studies.

Outcome	Present Study, % (95% CI)	Reference Study	Published Rate	Exploratory *p*
Device implantation	100 (96.4–100)	FLXibility [23]	99.0%	0.630
Device implantation	100 (96.4–100)	SURPASS [24]	97.5%	0.186
Any stroke at one year	1.0 (0.0–5.4)	FLXibility [23]	2.0%	0.727
Any stroke at one year	1.0 (0.0–5.4)	SURPASS [24]	1.5%	1.000
All-cause mortality at one year	11.0 (5.6–18.8)	FLXibility [23]	10.8%	0.873
All-cause mortality at one year	11.0 (5.6–18.8)	SURPASS [24]	8.2%	0.276

Present-study percentages use the original cohort of 100 patients; 95% CIs are exact binomial intervals. Reference values are the published percentages, rounded as reported. The *p*-values are two-sided exact binomial tests against those fixed percentages, without risk adjustment, reference-estimate uncertainty or correction for multiple comparisons. They are exploratory and are not formal between-cohort comparisons. The reference studies used their own endpoint definitions and follow-up denominators; their percentages are not necessarily calculated using the original enrolled cohort. All-stroke outcomes, rather than ischaemic-only or non-disabling-only stroke rates, are shown.

**Table 3:** Reported causes of death by follow-up period.

Cause of Death	0–30 Days *n*	31–365 Days *n*	Total *n*
**Cardiovascular deaths**	**0**	**2**	**2**
Decompensated heart failure	0	2	2
**Non-cardiovascular deaths**	**2**	**5**	**7**
Advanced urinary bladder cancer	1	0	1
End-stage renal failure	1	2	3
Sepsis	0	1	1
Acute renal failure	0	1	1
Pneumonia	0	1	1
**Unknown cause**	**0**	**2**	**2**
**All deaths**	**2**	**9**	**11**

Values are numbers of deaths in the original 100-patient cohort. Cardiovascular and non-cardiovascular rows are category subtotals; the diagnoses beneath each subtotal provide the breakdown. Unknown causes are not reclassified as non-cardiovascular. Pneumonia denotes a reported cause of death during follow-up, not a postoperative pneumonia endpoint.

## Data Availability

The data presented in this study are available from the corresponding author upon reasonable request. The data are not publicly available due to ethical restrictions and the protection of patient privacy in accordance with institutional policies and applicable data protection regulations.

## Abbreviations

**Abbreviation**	**Full Term**
AF	Atrial Fibrillation
BARC	Bleeding Academic Research Consortium
BMI	Body Mass Index
CAD	Coronary Artery Disease
CKD	Chronic Kidney Disease
DOAC	Direct Oral Anticoagulant
EHRA	European Heart Rhythm Association
EAPCI	European Association of Percutaneous Cardiovascular Interventions
HAS-BLED	Hypertension, Abnormal Renal/Liver Function, Stroke, Bleeding, Labile INR, Elderly, Drugs or Alcohol
LAA	Left Atrial Appendage
LAAO	Left Atrial Appendage Occlusion
NCDR	National Cardiovascular Data Registry
OAC	Oral Anticoagulation
PREVAIL	Prospective Randomised Evaluation of the Watchman Left Atrial Appendage Closure Device In Patients With Atrial Fibrillation Versus Long-Term Warfarin Therapy
PROTECT AF	Watchman Left Atrial Appendage System for Embolic Protection in Patients With Atrial Fibrillation
SD	Standard Deviation
TOE	Transoesophageal Echocardiography
VKA	Vitamin K Antagonist
